# Short stature-related factors and nomogram-based risk prediction in children aged 7-12: evidence from Chaozhou, China

**DOI:** 10.3389/fendo.2026.1598683

**Published:** 2026-02-20

**Authors:** Qun Zhang, Huarong Lin, Wencan Xu, Yifeng Cai

**Affiliations:** 1 Department of Endocrinology, Chaozhou Central Hospital, Chaozhou, China; 2 The First Affiliated Hospital of Shantou University Medical College, Shantou, China; 3 Department of Critical Care Medicine, Chaozhou Central Hospital, Chaozhou, China

**Keywords:** Chaozhou City, height development, risk factors, risk prediction model, short stature

## Abstract

**Objective:**

Childhood height development is a crucial indicator of public health, with the prevalence of short stature serving as an important metric. This study aimed to investigate the height development status, prevalence of short stature, and associated risk factors among 7-12-year-old children in Chaozhou City, China, providing valuable reference data for local prevention and intervention strategies to address short stature.

**Methods:**

A cross-sectional survey on the height of 7-12-year-old children was conducted in Chaozhou City, Guangdong Province, China. Standardized measurement tools were used to collect height data for epidemiological analysis. To explore risk factors for short stature, a questionnaire survey was administered to a random sample of the surveyed population. Univariate and multivariate logistic regression analyses were conducted to identify factors associated with the risk of short stature and to construct a predictive model.

**Results:**

A total of 7,799 children participated in the height survey. Girls had significantly higher mean heights than boys at ages 8, 11, and 12 (all *P* < 0.001). The overall prevalence of short stature was 3.7%. Although girls had a higher prevalence than boys (4.0% vs. 3.4%), the difference was not statistically significant (*P* = 0.167). Multivariate logistic regression identified independent risk factors for short stature, including paternal height < 160 cm, maternal height <150 cm, birth weight < 2.5 kg, preterm birth, exercising < 3 times per week, sleep duration < 8 hours per day, and irregular diet. A preference for meat and dairy products was independently associated with a reduced risk of short stature. The nomogram model developed based on these factors demonstrated good predictive performance, with an area under the curve of 0.858 (95%CI 0.815-0.900).

**Conclusions:**

The overall prevalence of short stature in 7-12-year-old children in Chaozhou was slightly higher than the national average. This study analyzed the risk factors for short stature in children, and the risk prediction model developed from these factors demonstrated good predictive accuracy for short stature prevalence. However, external validation in independent cohorts is necessary to confirm the robustness of the model.

## 1 Introduction

Linear growth in childhood is an important indicator of overall health, nutritional status, and living conditions. National surveillance data from the Chinese Center for Disease Control and Prevention indicate that, compared with 2012, the mean height of boys and girls aged 6–17 years increased by 1.6 cm and 1.0 cm, respectively (1). These improvements reflect China’s growing emphasis on child health and highlight the dynamic changes in growth patterns.

Despite these positive trends, short stature remains a public health concern in certain regions of China, where socioeconomic disparities, lifestyle differences, and uneven access to medical care may still influence childhood growth (2). A large national survey conducted in 2014 involving over 200,000 children reported age-standardized and age-sex-standardized prevalence rates of 3.70% and 2.69%, respectively, with clear regional variation (3). Moreover, China’s national height reference data, derived from large-scale surveys conducted in 2005 and growth curves established in 2009, have not been updated for more than a decade (4, 5). Given the country’s substantial geographic and developmental heterogeneity, updated region-specific data are needed to accurately characterize current growth patterns.

The etiology of short stature is multifactorial, involving nutritional, environmental, and psychosocial factors (6–10). However, marked regional differences in health awareness, medical resources, and lifestyle patterns suggest that risk factors and preventive strategies may vary across settings.

In Chaozhou, evidence on the prevalence and determinants of short stature remains scarce. To address this gap, we conducted a cross-sectional survey among children aged 7–12 years in urban Chaozhou to assess the current prevalence of short stature and its influencing factors, with the aim of providing updated evidence to support local and region-specific epublic health decision-making.

## 2 Methods

### 2.1 Study designs

A cross-sectional survey on height was conducted among children aged 7–12 in Chaozhou, Guangdong Province, between June and September 2023. This work was part of the 2023 Chaozhou Student Growth and Development Abnormality Monitoring and Intervention Project, jointly carried out by the Chaozhou Health Bureau, the Chaozhou Education Bureau, and local medical institutions. Height was measured for children of this age group using standardized tools. In addition, a random sample from the survey group was chosen for a questionnaire on short stature influencing factors.

### 2.2 Study participants

All children aged 7–12 were eligible for this study, except those with disabilities, congenital diseases, or unwillingness to participate. In the influencing factor survey, questionnaires with incomplete information or obvious logical errors were excluded.

### 2.3 Data collection

Height and weight measurements were taken by project members with standardized training. They used a calibrated stadiometer and weight scale (Brand: Ruke; Manufacturer: Wujin Instrument Co., Ltd.; Model: SZ-200; precision ±0.1 cm/kg). Children wore light clothing and stood barefoot with heels together and looking straight ahead. The measurers read the height at the bottom edge of the sliding board, with both height (cm) and weight (kg) recorded to one decimal place. Each measurement was taken three times and averaged. Data was double-checked and recorded in a summary table.

The questionnaire was developed online using the SoJump platform, a widely used online survey system in China, and was distributed to student guardians via QR code for completion. To investigate factors associated with short stature, stratified random sampling was applied using age and sex as key stratification variables. Subgroups were formed according to the proportional allocation principle, and approximately one-quarter of the population was randomly selected as the questionnaire sample. The electronic questionnaire was administered through an online platform, and all responses were verified by two independent reviewers with logic checks applied to ensure data quality. After collection, a third party uniformly exported, organized, and analyzed the final validated questionnaires.

The questionnaire collected information in the following domains: ①Basic demographic characteristics, including sex, age, height, weight, parental height, birth weight, and preterm birth status; ②Birth status and early-life factors, including birth weight, preterm birth status, feeding method during infancy; ③Lifestyle and physical activity, including daily sleep duration, bedtime, exercise frequency, weekly exercise duration and daily screen time; ④Dietary habits, including dietary regularity, food preferences, weekly snack consumption frequency, meal duration, and frequency of eating takeout per week.

### 2.4 Outcomes and definition

Short stature is defined as a height more than 2 standard deviations (SD) below the mean for children of the same age and sex, or below the 3rd percentile (P3, −1.88 SD), based on the Chinese national growth reference standards (11). This study used the percentile values of height and weight for Chinese children and adolescents aged 0–18 to screen for short stature (4, 5).

The primary endpoints were the height characteristics and prevalence of short stature among children aged 7–12 in Chaozhou. The secondary endpoints included the influencing factors of short stature and the construction of a predictive model.

### 2.5 Statistical analysis

The statistical analysis was performed using SPSS version 26.0 (IBM Corp., Armonk, NY, USA) and R version 4.2.2 (R Foundation for Statistical Computing, Vienna, Austria). Continuous variables were summarized as mean ± SD or median with interquartile range (IQR), whereas categorical variables were presented as frequencies and percentages. Differences between the height of children in Chaozhou and the 2005 national average were assessed using one-sample t-tests. Chi-square tests were used to compare the prevalence of short stature across genders and age groups. Height standard deviation scores (SDS) were calculated using age- and sex-specific Chinese growth standard curves (12), and are shown in **Supplementary Table 1**; **Supplementary Figure 1**.

Univariate logistic regression was conducted to screen potential factors associated with short stature, and variables with P < 0.10 were included in the multivariate logistic regression model to identify independent risk factors. A risk prediction model was developed based on significant predictors and visualized using a nomogram.

Model discrimination was evaluated using the area under the receiver operating characteristic (ROC) curve (AUC), with higher values indicating better discriminatory ability. An AUC > 0.7 is generally considered acceptable (13). Model calibration was assessed using calibration plots and the Hosmer-Lemeshow goodness-of-fit test, with P > 0.05 indicating adequate model calibration. A two-sided P < 0.05 was considered statistically significant.

### 2.6 Ethical considerations

Before formal measurements, researchers explained the study’s purpose and methods in detail to schools, parents, and students. Measurements were conducted and questionnaires were distributed only after obtaining guardians’ informed consent. To protect participants’ privacy, all survey information was strictly confidential. The study was approved by the Ethics Committee of the Chaozhou Central Hospital (approval No. 2023005).

## 3 Results

### 3.1 Study population and height characteristics

A total of 8,078 students aged 7–12 years were initially enrolled in Chaozhou, among whom 7,799 were included in the final analysis after excluding those with missing information or inability to cooperate. Of the analyzed population, 52.4% were male. The survey covered three administrative districts—Chao’an (25.5%), Raoping (30.2%), and Xiangqiao (44.3%). The age distribution for children aged 7 to 12 years was 17.9%, 15.9%, 16.4%, 17.3%, 18.4%, and 13.9%, respectively.wwc.

**Figure 1:**
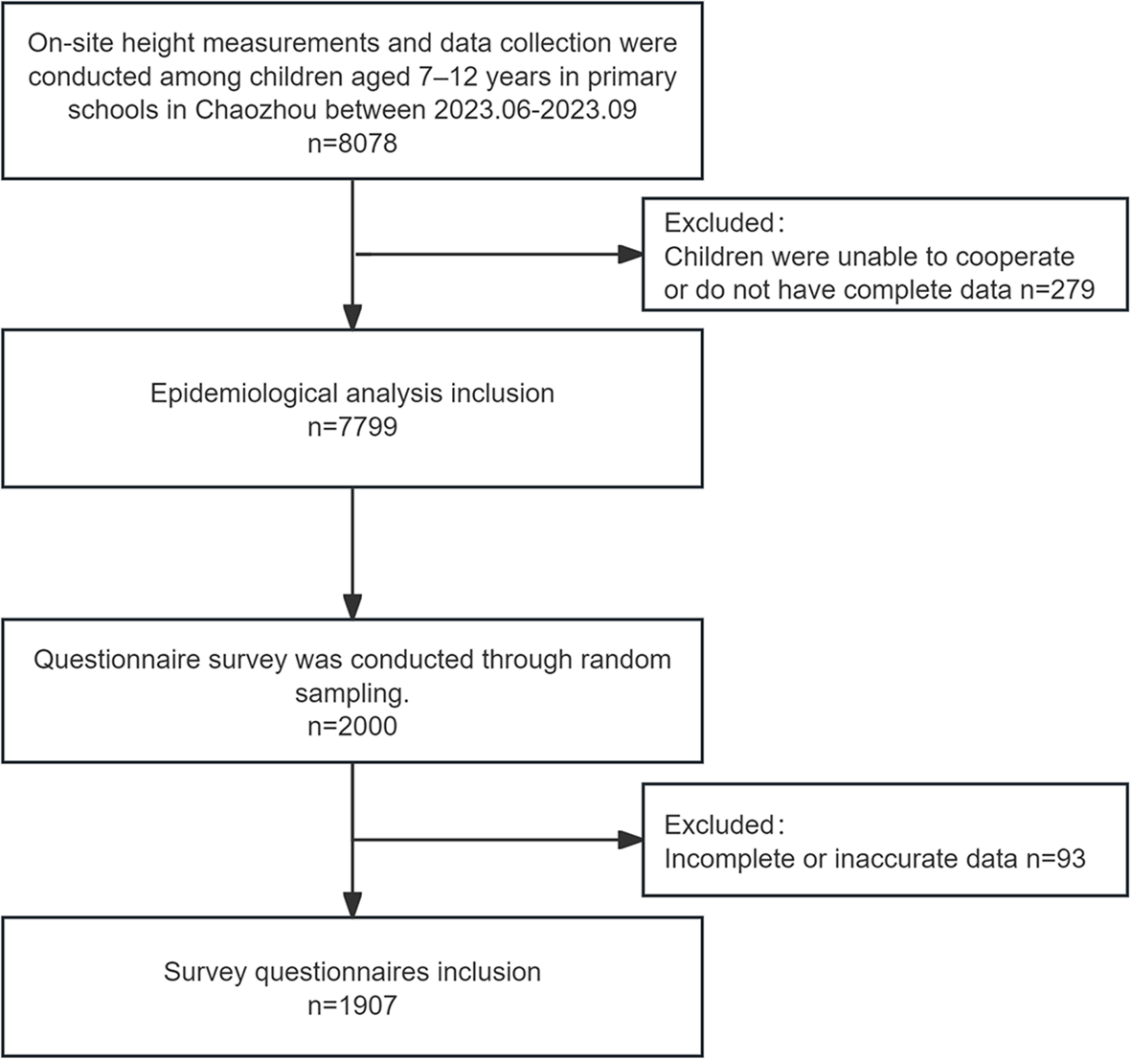
Flow chart illustrating the selection of patients.

**Figure 2:**
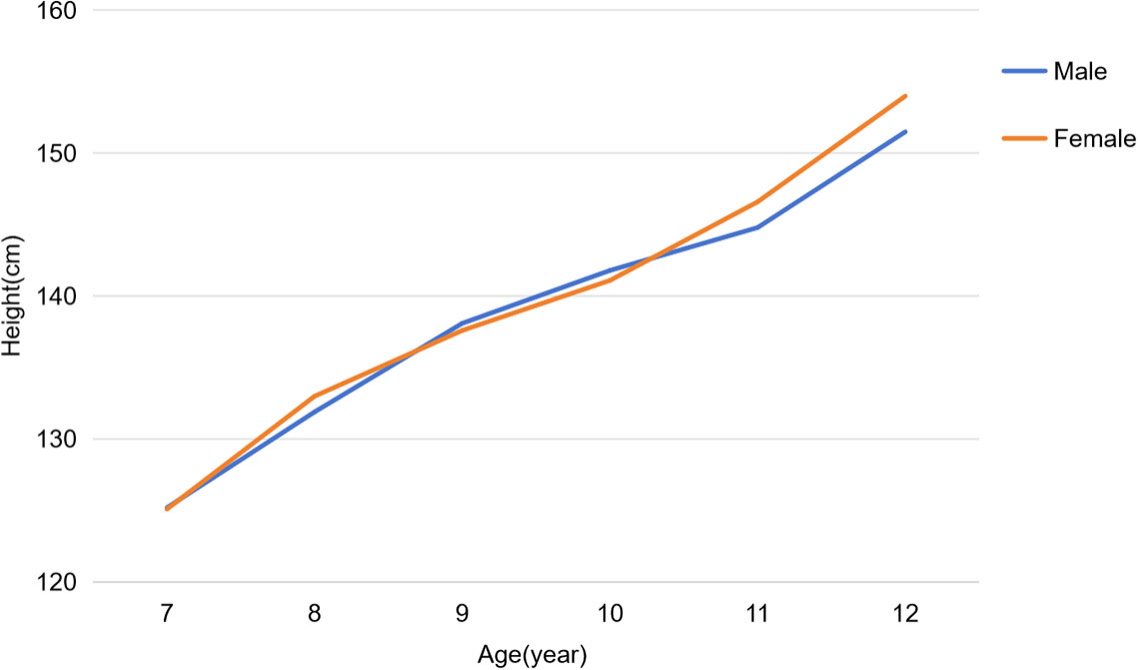
Height development characteristics of children aged 7–12 years.

Analysis of height by age and sex showed that girls had significantly greater mean height than boys at ages 8 (133.0 ± 3.8 cm vs. 131.9 ± 3.7 cm, P < 0.001), 11 (146.6 ± 9.7 cm vs. 144.8 ± 9.8 cm, P = 0.001), and 12 (154.0 ± 9.0 cm vs. 151.5 ± 9.3 cm, P < 0.001), while no significant differences were observed at other ages. The study flow is presented in **Figure 1**, and detailed demographic and height characteristics are summarized in **Table 1**; **Figure 2**.

**Table 1:** Height characteristics of children aged 7-12.

Age (year)	Proportion of males, n, (%)	Total height, cm, mean (SD)	Male height, cm, mean (SD)	Male mean height SDS (SD)	Female height, cm, mean (SD)	Female mean height SDS (SD)	*P* value
7	709 (50.7)	125.1 (5.3)	125.2 (4.7)	-0.37 (0.91)	125.1 (5.9)	-0.10 (1.13)	0.713
8	649 (52.3)	132.4 (3.8)	131.9 (3.7)	-0.15 (0.66)	133.0 (3.8)	0.31 (0.69)	< 0.001
9	655 (51.2)	137.8 (7.5)	138.1 (7.7)	0.04 (1.29)	137.6 (7.2)	0.09 (1.20)	0.186
10	721 (53.4)	141.5 (8.8)	141.8 (8.2)	-0.12 (1.42)	141.1 (8.7)	-0.34 (1.33)	0.142
11	825 (57.2)	145.6 (9.8)	144.8 (9.8)	-0.51 (1.40)	146.6 (9.7)	-0.46 (1.48)	0.001
12	530 (48.8)	152.8 (9.2)	151.5 (9.3)	-0.53 (1.20)	154.0 (9.0)	0.09 (1.45)	< 0.001

### 3.2 Prevalence of short stature in children aged 7-12

Among the 7,799 children included in this study, 287 were identified as having short stature, resulting in an overall prevalence of 3.7%. The prevalence of short stature growth among children aged 7 to 12 years was 2.2%, 1.8%, 4.4%, 7.3%, 8.4%, and 7.9%, respectively. There were significant differences in prevalence across age groups (χ² = 111.858, *P* < 0.001), with the highest prevalence observed in children aged 9–12 years.

In the overall population, the prevalence of short stature growth was 3.4% in boys and 4.0% in girls. Although the prevalence was higher in girls than in boys, the difference was not statistically significant (OR = 1.181, 95% CI: 0.933–1.495, *P* = 0.167). The prevalence among boys by age was 1.6%, 2.0%, 4.1%, 3.1%, 4.8%, and 4.9%, with a peak occurring between ages 9 and 12. Similarly, the prevalence among girls was 2.9%, 1.5%, 4.6%, 5.4%, 4.7%, and 4.9%, also peaking between ages 9 and 12.

**Figure 3:**
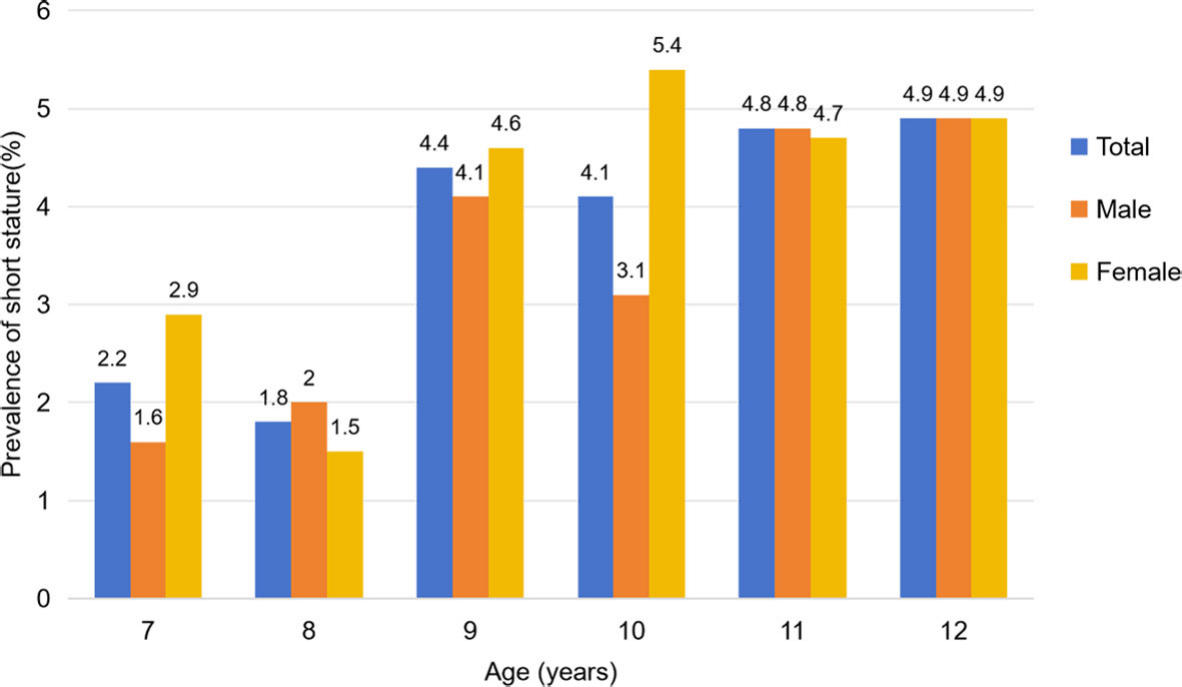
Prevalence of short stature in children aged 7–12 years.

At age 10, the prevalence of stunted growth was significantly higher in girls than in boys (OR = 1.813, 95% CI: 1.049–3.133, *P* = 0.031), while no statistically significant differences were observed between genders at other ages. Detailed data were presented in **Table 2**; **Figure 3**.

**Table 2:** Comparison of the prevalence of short stature across different age groups and the gender differences in prevalence.

Age (year)	Total	Male	Female	χ²	*P* value	OR	95%CI
7, n, (%)	31 (2.2)	11 (1.6)	20 (2.9)	2.929	0.087	1.894	0.901-3.983
8, n, (%)	22 (1.8)	12 (2.0)	9 (1.5)	0.414	0.520	0.755	0.320-1.780
9, n, (%)	56 (4.4)	27 (4.1)	29 (4.6)	0.205	0.651	1.132	0.662-1.934
10, n, (%)	56 (4.1)	22 (3.1)	34 (5.4)	4.655	0.031	1.813	1.049-3.133
11, n, (%)	69 (4.8)	40 (4.8)	29 (4.7)	0.017	0.896	0.968	0.593-1.580
12, n, (%)	53 (4.9)	26 (4.9)	27 (4.9)	0.001	0.970	0.989	0.570-1.719
Total, n, (%)	287 (3.7)	139 (3.4)	148 (4.0)	1.909	0.167	1.181	0.933-1.495

### 3.3 Predictors for short stature occurrence

In this study, 2000 questionnaires were randomly distributed, and 1907 valid ones were analyzed after excluding those with incomplete data or logical errors. Of these, 52.5% were from male respondents, and 4.9% reported short stature cases. Baseline data for the analyzed group are in **Table 3**.

**Table 3:** Analysis of factors related to short stature included baseline data from the study population.

Baseline characteristic	Total n = 1907	Baseline characteristic	Total n = 1907
Male, n, (%)	1001 (52.5)	Time to sleep [n, (%)]	
Short stature [n, (%)]	93 (4.9)	Before 21:00	489 (25.6)
Age [n, (%)]		21:00-23:00	1060 (55.6)
7	336 (17.6)	After 23:00	358 (18.8)
8	298 (15.6)	Irregular diet [n, (%)]	276 (14.5)
9	307 (16.1)	Food type preference [n, (%)]	
10	338 (17.7)	Preference for vegetables and fruits	610 (32.0)
11	389 (20.4)	Preference for meat and dairy	1148 (60.2)
12	239 (12.5)	Preference for grains	39 (20.7)
Father's height < 170 cm [n, (%)]	676 (35.4)	Snack consumption > 4 days per week [n, (%)]	855 (46.4)
Mother's height < 155cm [n, (%)]	337 (17.7)	Mealtime duration > 1 hour [n, (%)]	156 (8.2)
Birth weight < 4kg [n, (%)]	699 (36.7)	Ordering takeout > 3 times per week [n, (%)]	199 (10.4)
Prematurity [n, (%)]	171 (9.0)	Time of using phone on weekday [n, (%)]	
Feeding patterns [n, (%)]		< 1 hour	1461 (76.6)
Breast feeding	562 (29.5)	1-3hours	364 (19.1)
Bottle feeding	687 (36.0)	> 3 hours	82 (4.3)
Mixture feeding	658 (34.5)	Time of using phone on weekend [n, (%)]	
Exercising < 3 times per week [n, (%)]	612 (32.1)	< 1 hour	985 (31.7.6)
Exercising < 120 minutes per week [n, (%)]	613 (32.1)	1-3hours	695 (36.4)
Sleep duration < 8 hours [n, (%)]	248 (28.7)	> 3 hours	227 (11.9)

Univariate analysis showed that several factors were associated with short stature, including paternal height < 160 cm, maternal height < 150 cm, low birth weight (< 2.5 kg), preterm birth, exercising fewer than three times per week, weekly exercise time < 120 minutes, sleep duration < 8 hours, irregular eating patterns, preference for meat and dairy products, taking more than 1 hour per meal, and consuming takeout food more than three times per week.

Variables with P < 0.10 were entered into a multivariate logistic regression model. The results indicated that paternal height < 160 cm (OR = 10.677, 95% CI: 5.694-20.022), maternal height < 150 cm (OR = 8.071, 95% CI: 3.975-16.389), birth weight < 2.5 kg (OR = 8.750, 95% CI: 4.210-18.185), preterm birth (OR = 3.724, 95% CI: 2.012-6.891), exercising fewer than three times per week (OR = 2.815, 95% CI: 1.682-4.711), sleep duration <8 hours (OR = 2.294, 95% CI: 1.352-3.892), and irregular diet (OR = 3.264, 95% CI: 1.837-5.801) were independent risk factors for short stature. Conversely, a preference for meat and dairy products was independently associated with a reduced risk (OR = 0.519, 95% CI: 0.303-0.888). Details are shown in **Table 4**.

**Table 4:** Univariate and multivariate analysis of factors associated with short stature.

	Univariate analysis			Multivariate analysis		
	OR	95% CI	P	OR	95% CI	P
Father's height < 170 cm [n, (%)]	0.906	0.583-1.409	0.662			
Mother's height < 155cm [n, (%)]	0.891	0.506-1.569	0.689			
Birth weight < 4kg [n, (%)]	3.343	2.163-5.168	<0.001	3.455	2.163-5.521	<0.001
Prematurity [n, (%)]	2.224	1.266-3.906	0.005	2.063	1.073-3.968	0.030
Feeding patterns [n, (%)]			0.926			
Bottle feeding vs. Breast feeding	1.221	0.735-2.028	0.440			
Mixture feeding vs. Breast feeding	0.924	0.553-1.544	0.762			
Exercising < 3 times per week [n, (%)]	1.989	1.302-3.039	0.001	2.589	1.617-4.148	<0.001
Exercising < 120 minutes per week [n, (%)]	1.493	0.976-2.283	0.065	1.587	0.989-2.546	0.056
Sleep duration < 8 hours [n, (%)]	2.935	1.928-4.466	<0.001	5.19	3.032-8.884	<0.001
Time to sleep [n, (%)]						
21:00-23:00 vs. Before 21:00	0.674	0.321-1.415	0.297			
After 23:00 vs. Before 21:00	1.469	0.826-2.612	0.190			
Irregular diet [n, (%)]	2.571	1.611-4.103	<0.001	4.022	2.327-6.952	<0.001
Food type preference [n, (%)]						
Preference for vegetables and fruits	1.364	0.889-2.095	0.156			
Preference for meat and dairy	0.399	0.26-0.612	<0.001	0.31	0.184-0.522	<0.001
Preference for grains	0.611	0.336-1.11	0.106			
Snack consumption > 4 days per week [n, (%)]	1.303	0.858-1.978	0.214			
Mealtime duration > 1 hour [n, (%)]	2.282	1.279-4.07	0.005	1.615	0.8-3.261	0.181
Ordering takeout > 3 times per week [n, (%)]	2.331	1.376-3.948	0.002	1.359	0.715-2.581	0.349
Time of using phone on weekday [n, (%)]	2.156	1.597-2.912	<0.001	3.128	2.158-4.534	<0.001
Time of using phone on weekend [n, (%)]	1.336	1.004-1.779	0.047	2.317	1.602-3.35	<0.001

### 3.4 Nomogram model development and evaluation

**Figure 4:**
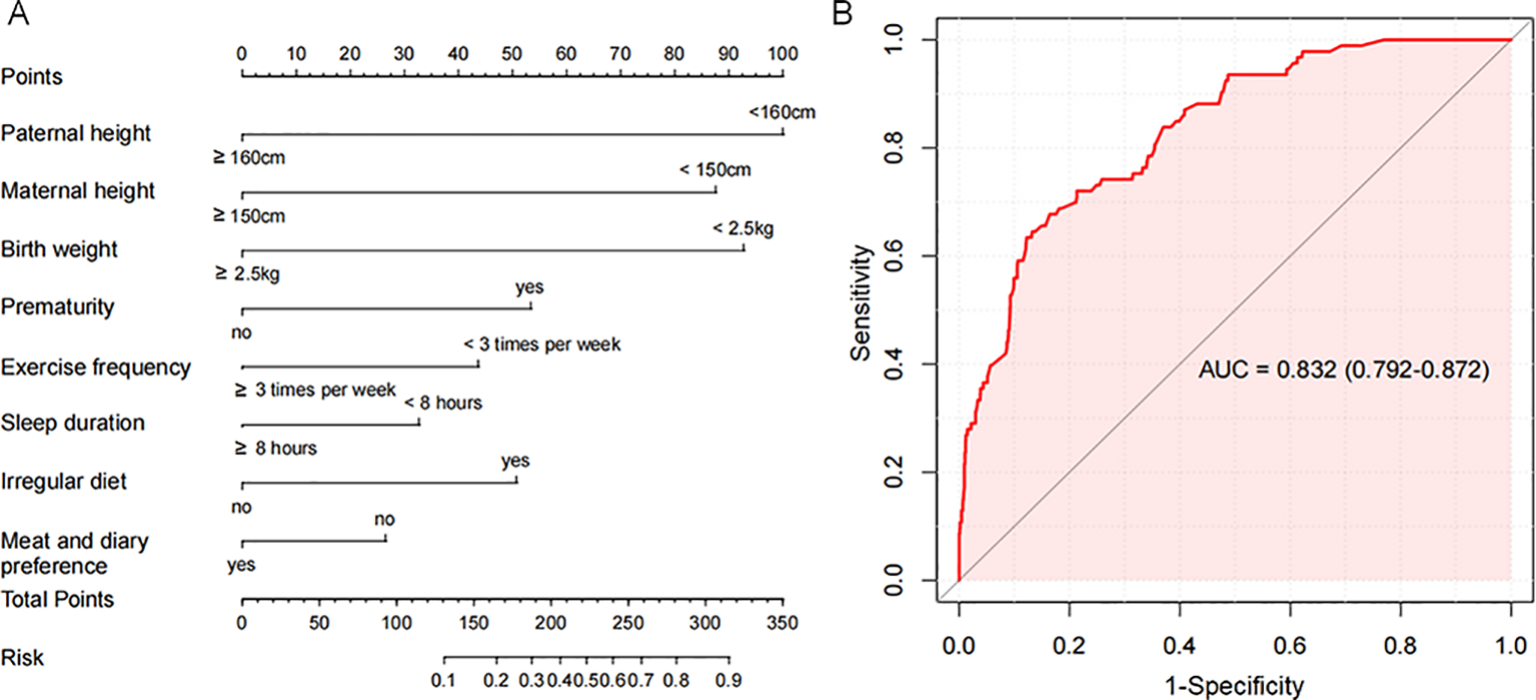
Nomogram model for predicting short stature. **(A)** A nomogram model constructed based on eight identified risk factors for short stature. **(B)** Area under the ROC of the nomogram model.

A nomogram model was developed based on the eight identified risk factors (**Figure 4a**). The diagnostic equation derived from multivariable logistic regression was: ln(p/(1-p)) = 2.3839 × paternal height + 2.0875 × maternal height + 2.2107 × birth weight + 1.2718 × prematurity + 1.0400 × weekly exercise frequency + 0.7789 × sleep duration + 1.2085 × regular diet − 0.6314 × preference for meat/dairy − 4.6828. All predictors were entered as binary variables, coded as follows: paternal height (<160 cm = 1, ≥160 cm = 0), maternal height (<150 cm = 1, ≥150 cm = 0), birth weight (<2.5 kg = 1, ≥2.5 kg = 0), prematurity (yes = 1, no = 0), weekly exercise frequency (<3 times/week = 1, ≥3 times/week = 0), sleep duration (<8 h = 1, ≥8 h = 0), regular diet (no = 1, yes = 0), and preference for meat/dairy (no = 1, yes = 0).

**Figure 5:**
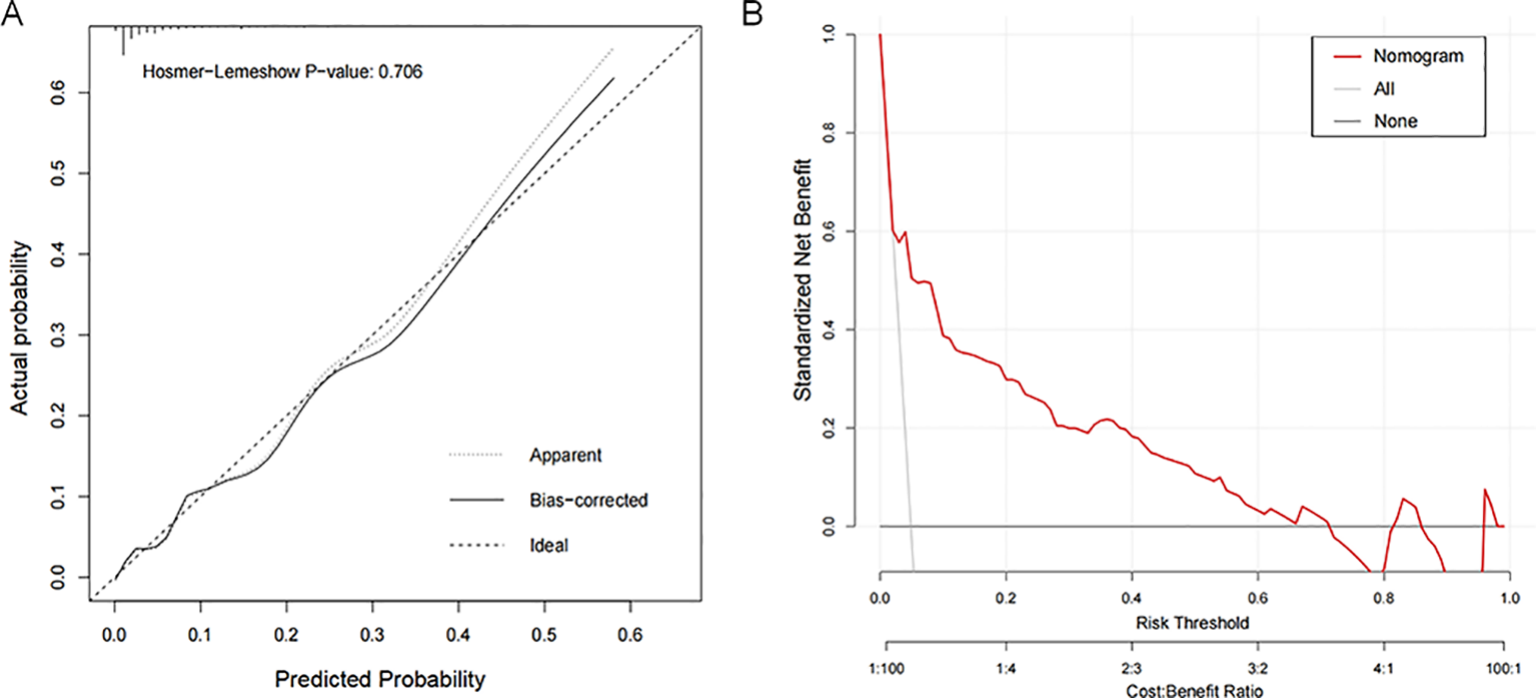
Evaluation of nomogram prediction models. **(A)** Calibration assessed by the Hosmer-Lemeshow test; **(B)** The calibration curve of the nomogram model.

The AUC of the nomogram model for predicting short stature occurrence was 0.858 (95% CI: 0.815-0.900), which is shown in **Figure 4b**. Calibration assessed by the Hosmer–Lemeshow test showed no significant deviation between predicted and observed probabilities (P = 0.104), indicating satisfactory fit (**Figure 5a**). Decision curve analysis further showed that the nomogram provided greater net clinical benefit across a broad range of threshold probabilities compared with strategies assuming all or no children at risk, supporting its value in short stature risk prediction and clinical decision-making. The DCA curve is displayed in **Figure 5b**.

## 4 Disscussion

In this population-based study of children aged 7–12 years in Chaozhou, China, we investigated the occurrence of short stature and the related factors within a regional context. The prevalence of short stature in the study population was 3.7%, which is slightly lower than the national average reported in previous studies. We found that the occurrence of short stature in this area was influenced by a combination of familial background, perinatal conditions, and modifiable lifestyle-related factors, emphasizing that the factors influencing short stature are multifaceted.

In our study, the mean height of girls at 8, 11, and 12 years exceeded that of boys, with no significant sex differences observed at other ages. This pattern is consistent with the earlier onset of puberty in girls (14). At 11–12 ages, many girls are approaching or have reached peak height velocity, whereas boys typically remain in an earlier stage of growth (15, 16). Interestingly, we observed a slight height advantage in girls even at age 8. This stage, which typically occurs around 7–9 years of age, precedes the true pubertal growth spurt and is influenced in part by early maturation processes such as adrenarche. As girls generally mature earlier than boys by approximately 1–2 years, this modest increase in growth velocity can result in a subtle height advantage even before the onset of overt puberty (17). Additionally, local population characteristics or sampling variation may also contribute to this observation. Nevertheless, although statistically significant, the magnitude of the height difference at age 8 is small and its clinical significance is limited. Future studies incorporating bone age and pubertal development assessments may help further elucidate whether this pattern reflects normal sex-specific growth timing or region-specific growth dynamics.

The prevalence of short stature provides region-specific public health information. A nationwide meta-analysis of 39 studies involving 348326 children reported a prevalence of 3.2% (95% CI: 2.6-3.7%) in China, with no significant sex difference (3.1% in boys vs. 3.2% in girls, P > 0.05). Notably, substantial regional variation was observed, ranging from 5.2% (95% CI: 4.4-6.0%) in western China to 0.6% (95% CI: 0.3-0.8%) in northern areas (18). In the Chaozhou cohort, the prevalence was 3.7%, slightly higher than the national level, and showed no sex disparity. Age-stratified analysis revealed a peak between 9 and 12 years, whereas children aged 7–8 demonstrated lower prevalence than nationwide estimates. These results suggest that children entering puberty represent a critical developmental stage, underscoring the need for enhanced monitoring, nutritional support, and targeted health education during this period.

We found that parental height was strongly associated with the occurrence of short stature. Parental stature likely reflects inherited genetic influences on growth-plate biology and longitudinal growth potential (19). Consistent with this, a large trans-ancestry longitudinal genome-wide association study has shown that genetic variation plays a key role in determining the timing, velocity, and overall magnitude of height growth during puberty (20). Furthermore, the heritability of height increases from early childhood into adolescence, indicating that parental genetic background becomes progressively more influential in determining final stature (21).

Birth-related conditions was another significant factors. Low birth weight and preterm birth often reflect intrauterine growth restriction, which may impair both neonatal size and later growth potential. A pooled analysis of 19 birth cohorts showed that preterm (OR = 1.93), small for gestational age (SGA) infants (OR = 2.43), and preterm-SGA (OR = 4.51) infants had significantly increased risks of developmental delay compared with term, appropriate-for-gestational-age infants (22). Furthermore, preterm birth may also cause metabolic and endocrine disturbances resembling anterior pituitary hypofunction, featuring low IGF-1 levels, increased fat accumulation, and poor linear growth (23, 24). In SGA infants, adequate catch-up growth is essential to compensate for fetal growth restriction; however, children with persistent nutritional or metabolic insufficiency may fail to catch up, resulting in sustained height deficits later in life (25).

Lifestyle factors are also important for growth in children. In our study, moderate physical activity was associated with a lower risk of short stature, whereas insufficient sleep of less than eight hours per night was identified as an independent risk factor. Sleep and exercise both support normal development. Poor sleep may disturb circadian rhythms, affect the hypothalamic pituitary growth hormone axis, and lead to metabolic imbalance, which can hinder height growth (26–28). Furthermore, dietary habits showed a similar impact. Irregular diets may reduce total energy intake and lead to deficiencies in protein, vitamins, and minerals that are necessary for normal growth (29). While animal-source foods contained high-quality protein, iron, zinc, and vitamin B12 promote skeletal and linear growth (30). A multinational study and a systematic review both reported that meat consumption is linked to better growth and lower developmental delay (31, 32).

Huang et al. analyzed risk factors for short stature among 1,076 children aged 6–12 years in Shenzhen and identified parental height, household income, paternal education level, and parental concern for height as significant determinants. Based on these variables, a nomogram prediction model was developed with an AUC of 0.748, and a total score of 127.5 served as the cut-off for risk stratification (9). Similarly, we constructed a predictive model incorporating eight independent factors and visualized it using a nomogram. Our model achieved an AUC > 0.8 with good calibration, indicating strong predictive performance. However, as both development and validation were conducted internally within a single regional cohort, external and prospective validation remains necessary.

Our study has notable strengths. It represents the largest recent epidemiological investigation of short stature among children aged 7–12 years in Chaozhou and includes nearly 8,000 participants, making the findings highly representative. We also analyzed social and environmental determinants through questionnaires and established a risk prediction model with a nomogram to support clinical use, which showed good internal accuracy and calibration. However, several limitations exist. As a cross-sectional survey conducted in Chaozhou, the results mainly reflect local growth patterns and may not fully apply to other regions. The use of self-reported questionnaires may introduce reporting bias despite careful design and data screening. In addition, only social and environmental factors were assessed, while genetic and biological factors were not included. Causal inference cannot be drawn due to the study design, and external validation is needed to confirm the model’s generalizability.

In summary, this study outlines height status, short-stature prevalence, and related social and environmental factors among children aged 7–12 in Chaozhou, providing evidence for policymaking and early intervention. Future prospective studies are needed to clarify influencing factors and support more effective growth management strategies.

## Data Availability

The original contributions presented in the study are included in the article/**Supplementary Material**. Further inquiries can be directed to the corresponding author.
