# Supplementary material for: Short stature-related factors and nomogram-based risk prediction in children aged 7-12: evidence from Chaozhou, China
Source: Front Endocrinol (Lausanne). 2026 Feb 20;17:1598683. doi: 10.3389/fendo.2026.1598683 (PMC12962942; doi:10.3389/fendo.2026.1598683)

**Supplementary materials**

**Supplementary document**

**Supplementary Table 1.** Height reference standards for Chinese children (7-12 years).

**Supplementary Figure 1.** Growth Curve Chart of Height and Weight Standard Deviation Units for Chinese Boys Aged 2–18 Years.

**Supplementary Figure 2.** Growth Curve Chart of Height and Weight Standard Deviation Units for Chinese Girls Aged 2–18 Years.

**Supplementary Table 1.** Height reference standards for Chinese children (7-12 years).

| Age | SD-corresponding Height (cm) for boys | | | | | | |  | SD-corresponding Height (cm) for girls | | | | | | |
| --- | --- | --- | --- | --- | --- | --- | --- | --- | --- | --- | --- | --- | --- | --- | --- |
|  | -3SD | -2SD | -1SD | Median | 1SD | 2SD | 3SD |  | -3SD | -2SD | -1SD | Median | 1SD | 2SD | 3SD |
| 7y | 109.2 | 114.0 | 119.0 | 124.0 | 129.1 | 134.3 | 139.6 |  | 108.0 | 112.7 | 117.6 | 122.5 | 127.6 | 132.7 | 137.9 |
| 7y1m | 109.6 | 114.5 | 119.5 | 124.5 | 129.7 | 134.9 | 140.2 |  | 108.4 | 113.2 | 118.1 | 123.0 | 128.1 | 133.3 | 138.5 |
| 7y2m | 110.1 | 114.9 | 120.0 | 125.0 | 130.2 | 135.5 | 140.9 |  | 108.8 | 113.6 | 118.5 | 123.5 | 128.7 | 133.8 | 139.1 |
| 7y3m | 110.5 | 115.4 | 120.5 | 125.6 | 130.8 | 136.1 | 141.5 |  | 109.2 | 114.1 | 119.0 | 124.1 | 129.2 | 134.4 | 139.7 |
| 7y4m | 110.9 | 115.9 | 120.9 | 126.1 | 131.3 | 136.6 | 142.1 |  | 109.6 | 114.5 | 119.5 | 124.6 | 129.7 | 135.0 | 140.3 |
| 7y5m | 111.4 | 116.3 | 121.4 | 126.6 | 131.9 | 137.2 | 142.8 |  | 110.0 | 115.0 | 119.9 | 125.1 | 130.3 | 135.5 | 140.9 |
| 7.5y | 111.8 | 116.8 | 121.9 | 127.1 | 132.4 | 137.8 | 143.4 |  | 110.4 | 115.4 | 120.4 | 125.6 | 130.8 | 136.1 | 141.5 |
| 7y7m | 112.2 | 117.2 | 122.4 | 127.6 | 132.9 | 138.4 | 144.0 |  | 110.8 | 115.8 | 120.9 | 126.1 | 131.3 | 136.7 | 142.1 |
| 7y8m | 112.6 | 117.6 | 122.8 | 128.1 | 133.4 | 138.9 | 144.5 |  | 111.2 | 116.2 | 121.3 | 126.6 | 131.8 | 137.2 | 142.6 |
| 7y9m | 113.0 | 118.1 | 123.3 | 128.6 | 134.0 | 139.5 | 145.1 |  | 111.6 | 116.7 | 121.8 | 127.1 | 132.4 | 137.8 | 143.2 |
| 7y10m | 113.3 | 118.5 | 123.7 | 129.0 | 134.5 | 140.0 | 145.7 |  | 111.9 | 117.1 | 122.2 | 127.5 | 132.9 | 138.3 | 143.8 |
| 7y11m | 113.7 | 118.9 | 124.2 | 129.5 | 135.0 | 140.6 | 146.2 |  | 112.3 | 117.5 | 122.7 | 128.0 | 133.4 | 138.9 | 144.3 |
| 8y | 114.1 | 119.3 | 124.6 | 130.0 | 135.5 | 141.1 | 146.8 |  | 112.7 | 117.9 | 123.1 | 128.5 | 133.9 | 139.4 | 144.9 |
| 8y1m | 114.5 | 119.7 | 125.0 | 130.5 | 136.0 | 141.6 | 147.4 |  | 113.1 | 118.3 | 123.6 | 129.0 | 134.4 | 139.9 | 145.5 |
| 8y2m | 114.8 | 120.1 | 125.4 | 131.0 | 136.5 | 142.1 | 147.9 |  | 113.5 | 118.7 | 124.0 | 129.4 | 134.9 | 140.5 | 146.1 |
| 8y3m | 115.2 | 120.5 | 125.9 | 131.4 | 137.0 | 142.7 | 148.5 |  | 113.9 | 119.1 | 124.5 | 129.9 | 135.4 | 141.0 | 146.7 |
| 8y4m | 115.5 | 120.8 | 126.3 | 131.8 | 137.4 | 143.2 | 149.0 |  | 114.2 | 119.5 | 124.9 | 130.4 | 135.9 | 141.5 | 147.2 |
| 8y5m | 115.9 | 121.2 | 126.7 | 132.3 | 137.9 | 143.7 | 149.6 |  | 114.6 | 119.9 | 125.4 | 130.8 | 136.4 | 142.1 | 147.8 |
| 8.5y | 116.2 | 121.6 | 127.1 | 132.7 | 138.4 | 144.2 | 150.1 |  | 115.0 | 120.3 | 125.8 | 131.3 | 136.9 | 142.6 | 148.4 |
| 8.7y | 116.6 | 122.0 | 127.5 | 133.2 | 138.9 | 144.7 | 150.6 |  | 115.3 | 120.7 | 126.2 | 131.8 | 137.4 | 143.1 | 149.0 |
| 8.8y | 116.9 | 122.4 | 127.9 | 133.6 | 139.3 | 145.2 | 151.2 |  | 115.7 | 121.1 | 126.6 | 132.2 | 137.9 | 143.7 | 149.5 |
| 8.9y | 117.3 | 122.8 | 128.4 | 134.1 | 139.8 | 145.7 | 151.7 |  | 116.0 | 121.5 | 127.1 | 132.7 | 138.4 | 144.2 | 150.1 |
| 8.10y | 117.6 | 123.1 | 128.8 | 134.5 | 140.3 | 146.2 | 152.2 |  | 116.3 | 121.8 | 127.5 | 133.2 | 138.9 | 144.7 | 150.7 |
| 8.11y | 118.0 | 123.5 | 129.2 | 135.0 | 140.7 | 146.7 | 152.8 |  | 116.7 | 122.2 | 127.9 | 133.6 | 139.4 | 145.3 | 151.2 |
| 9y | 118.3 | 123.9 | 129.6 | 135.4 | 141.2 | 147.2 | 153.3 |  | 117.0 | 122.6 | 128.3 | 134.1 | 139.9 | 145.8 | 151.8 |
| 9y1m | 118.6 | 124.3 | 130.0 | 135.8 | 141.7 | 147.7 | 153.8 |  | 117.4 | 123.0 | 128.8 | 134.6 | 140.4 | 146.4 | 152.4 |
| 9y2m | 119.0 | 124.6 | 130.4 | 136.2 | 142.1 | 148.2 | 154.3 |  | 117.7 | 123.4 | 129.2 | 135.1 | 141.0 | 146.9 | 153.0 |
| 9y3m | 119.3 | 125.0 | 130.8 | 136.7 | 142.6 | 148.7 | 154.9 |  | 118.1 | 123.8 | 129.7 | 135.6 | 141.5 | 147.5 | 153.6 |
| 9y4m | 119.6 | 125.3 | 131.1 | 137.1 | 143.1 | 149.1 | 155.4 |  | 118.4 | 124.2 | 130.1 | 136.0 | 142.0 | 148.1 | 154.2 |
| 9y5m | 120.0 | 125.7 | 131.5 | 137.5 | 143.5 | 149.6 | 155.9 |  | 118.8 | 124.6 | 130.6 | 136.5 | 142.6 | 148.6 | 154.8 |
| 9.5y | 120.3 | 126.0 | 131.9 | 137.9 | 144.0 | 150.1 | 156.4 |  | 119.1 | 125.0 | 131.0 | 137.0 | 143.1 | 149.2 | 155.4 |
| 9y7m | 120.6 | 126.3 | 132.3 | 138.3 | 144.4 | 150.5 | 156.9 |  | 119.5 | 125.4 | 131.5 | 137.5 | 143.7 | 149.8 | 156.0 |
| 9y8m | 120.9 | 126.6 | 132.6 | 138.7 | 144.8 | 151.0 | 157.3 |  | 119.9 | 125.9 | 131.9 | 138.0 | 144.2 | 150.4 | 156.7 |
| 9y9m | 121.2 | 127.0 | 133.0 | 139.1 | 145.2 | 151.4 | 157.8 |  | 120.3 | 126.3 | 132.4 | 138.6 | 144.8 | 151.0 | 157.3 |
| 9y10m | 121.4 | 127.3 | 133.3 | 139.4 | 145.6 | 151.8 | 158.3 |  | 120.7 | 126.7 | 132.9 | 139.1 | 145.3 | 151.6 | 157.9 |
| 9y11m | 121.7 | 127.6 | 133.7 | 139.8 | 146.0 | 152.3 | 158.7 |  | 121.1 | 127.2 | 133.3 | 139.6 | 145.9 | 152.2 | 158.6 |
| 10y | 122.0 | 127.9 | 134.0 | 140.2 | 146.4 | 152.7 | 159.2 |  | 121.5 | 127.6 | 133.8 | 140.1 | 146.4 | 152.8 | 159.2 |
| 10y1m | 122.3 | 128.3 | 134.4 | 140.6 | 146.9 | 153.2 | 159.7 |  | 121.9 | 128.1 | 134.3 | 140.6 | 147.0 | 153.4 | 159.8 |
| 10y2m | 122.6 | 128.6 | 134.8 | 141.0 | 147.3 | 153.7 | 160.2 |  | 122.3 | 128.5 | 134.8 | 141.2 | 147.6 | 154.0 | 160.5 |
| 10y3m | 122.9 | 129.0 | 135.2 | 141.4 | 147.8 | 154.2 | 160.8 |  | 122.7 | 129.0 | 135.3 | 141.7 | 148.1 | 154.6 | 161.1 |
| 10y4m | 123.2 | 129.3 | 135.5 | 141.8 | 148.2 | 154.7 | 161.3 |  | 123.1 | 129.4 | 135.8 | 142.2 | 148.7 | 155.1 | 161.7 |
| 10y5m | 123.5 | 129.7 | 135.9 | 142.2 | 148.6 | 155.2 | 161.8 |  | 123.5 | 129.9 | 136.3 | 142.8 | 149.2 | 155.7 | 162.4 |
| 10.5y | 123.8 | 130.0 | 136.3 | 142.6 | 149.1 | 155.7 | 162.3 |  | 123.9 | 130.3 | 136.8 | 143.3 | 149.8 | 156.3 | 163.0 |
| 10y7m | 124.1 | 130.4 | 136.7 | 143.1 | 149.6 | 156.2 | 162.9 |  | 124.4 | 130.8 | 137.3 | 143.9 | 150.4 | 156.9 | 163.6 |
| 10y8m | 124.4 | 130.7 | 137.1 | 143.5 | 150.1 | 156.8 | 163.5 |  | 124.9 | 131.3 | 137.9 | 144.4 | 151.0 | 157.5 | 164.2 |
| 10y9m | 124.8 | 131.1 | 137.5 | 144.0 | 150.6 | 157.3 | 164.1 |  | 125.4 | 131.9 | 138.4 | 145.0 | 151.6 | 158.2 | 164.9 |
| 10y10m | 125.1 | 131.4 | 137.9 | 144.4 | 151.1 | 157.8 | 164.6 |  | 125.9 | 132.4 | 138.9 | 145.5 | 152.1 | 158.8 | 165.5 |
| 10y11m | 125.4 | 131.8 | 138.3 | 144.9 | 151.6 | 158.4 | 165.2 |  | 126.4 | 132.9 | 139.5 | 146.1 | 152.7 | 159.4 | 166.1 |
| 11y | 125.7 | 132.1 | 138.7 | 145.3 | 152.1 | 158.9 | 165.8 |  | 126.9 | 133.4 | 140.0 | 146.6 | 153.3 | 160.0 | 166.7 |
| 11y1m | 126.0 | 132.5 | 139.2 | 145.8 | 152.7 | 159.5 | 166.5 |  | 127.4 | 133.9 | 140.5 | 147.1 | 153.8 | 160.5 | 167.2 |
| 11y2m | 126.4 | 132.9 | 139.6 | 146.3 | 153.2 | 160.1 | 167.1 |  | 127.9 | 134.4 | 141.0 | 147.6 | 154.3 | 161.1 | 167.7 |
| 11y3m | 126.7 | 133.3 | 140.1 | 146.9 | 153.8 | 160.8 | 167.8 |  | 128.4 | 135.0 | 141.6 | 148.2 | 154.8 | 161.5 | 168.2 |
| 11y4m | 127.0 | 133.7 | 140.6 | 147.4 | 154.3 | 161.4 | 168.5 |  | 128.9 | 135.5 | 142.1 | 148.7 | 155.3 | 161.9 | 168.6 |
| 11y5m | 127.4 | 134.1 | 141.0 | 147.9 | 154.9 | 162.0 | 169.1 |  | 129.4 | 136.0 | 142.6 | 149.2 | 155.8 | 162.4 | 169.1 |
| 11y6m | 127.7 | 134.5 | 141.4 | 148.4 | 155.4 | 162.6 | 169.8 |  | 129.9 | 136.5 | 143.1 | 149.7 | 156.3 | 162.9 | 169.6 |
| 11y7m | 128.1 | 135.0 | 141.9 | 149.0 | 156.1 | 163.3 | 170.6 |  | 130.4 | 137.0 | 143.6 | 150.2 | 156.7 | 163.3 | 170.0 |
| 11y8m | 128.5 | 135.4 | 142.5 | 149.6 | 156.7 | 164.0 | 171.4 |  | 130.9 | 137.5 | 144.0 | 150.6 | 157.1 | 163.7 | 170.3 |
| 11y9m | 128.9 | 135.9 | 143.0 | 150.2 | 157.4 | 164.8 | 172.2 |  | 131.5 | 138.0 | 144.5 | 151.1 | 157.6 | 164.1 | 170.7 |
| 11y10m | 129.2 | 136.3 | 143.5 | 150.7 | 158.1 | 165.5 | 172.9 |  | 132.0 | 138.5 | 145.0 | 151.7 | 158.0 | 164.5 | 171.1 |
| 11y11m | 129.6 | 136.8 | 144.1 | 151.3 | 158.7 | 166.2 | 173.7 |  | 132.5 | 139.0 | 145.4 | 152.0 | 158.4 | 164.9 | 171.4 |
| 12y | 130.0 | 137.2 | 144.6 | 151.9 | 159.4 | 166.9 | 174.5 |  | 133.0 | 139.5 | 145.9 | 152.4 | 158.8 | 165.3 | 171.8 |
| 12y1m | 130.4 | 137.7 | 145.2 | 152.5 | 160.1 | 167.9 | 175.2 |  | 133.5 | 139.9 | 146.3 | 152.8 | 159.1 | 165.6 | 172.1 |
| 12y2m | 130.9 | 138.2 | 145.7 | 153.1 | 160.7 | 168.3 | 176.0 |  | 134.0 | 140.4 | 146.7 | 153.1 | 159.5 | 165.9 | 172.3 |
| 12y3m | 131.3 | 138.7 | 146.3 | 153.8 | 161.4 | 169.0 | 176.7 |  | 134.5 | 140.8 | 147.2 | 153.5 | 159.8 | 166.2 | 172.6 |
| 12y4m | 131.7 | 139.2 | 146.8 | 154.4 | 162.0 | 169.7 | 177.4 |  | 134.9 | 141.2 | 147.6 | 153.9 | 160.1 | 166.5 | 172.8 |
| 12y5m | 132.2 | 139.7 | 147.4 | 155.0 | 162.7 | 170.4 | 178.2 |  | 135.4 | 141.7 | 148.0 | 154.2 | 160.5 | 166.8 | 173.1 |
| 12.5y | 132.6 | 140.2 | 147.9 | 155.6 | 163.3 | 171.1 | 178.9 |  | 135.9 | 142.1 | 148.4 | 154.6 | 160.8 | 167.1 | 173.3 |
| 12y7m | 133.2 | 140.8 | 148.6 | 156.3 | 164.0 | 171.8 | 179.6 |  | 136.3 | 142.5 | 148.7 | 154.9 | 161.1 | 167.3 | 173.5 |
| 12y8m | 133.8 | 141.5 | 149.2 | 156.9 | 164.6 | 172.4 | 180.3 |  | 136.7 | 142.8 | 149.0 | 155.2 | 161.3 | 167.5 | 173.6 |
| 12y9m | 134.5 | 142.1 | 149.9 | 157.6 | 165.3 | 173.1 | 181.0 |  | 137.1 | 143.2 | 149.4 | 155.5 | 161.6 | 167.7 | 173.8 |
| 12y10m | 135.1 | 142.7 | 150.5 | 158.2 | 166.0 | 173.8 | 181.6 |  | 137.4 | 143.5 | 149.7 | 155.7 | 161.8 | 167.9 | 174.0 |
| 12y11m | 135.7 | 143.4 | 151.2 | 158.9 | 166.6 | 174.4 | 182.3 |  | 137.7 | 143.9 | 150.0 | 156.0 | 162.1 | 168.1 | 174.1 |

**Source：**The Subspecialty Group of Endocrinologic, Hereditary and Metabolic Diseases, the Society of Pediatrics, Chinese Medical Association. (2009). Growth standard for Chinese children and adolescents (0–18 years). Chinese Journal of Pediatrics, 47(7): 487-492.

**Supplementary Figure 1.** Growth Curve Chart of Height and Weight Standard Deviation Units for Chinese Boys Aged 2–18 Years.


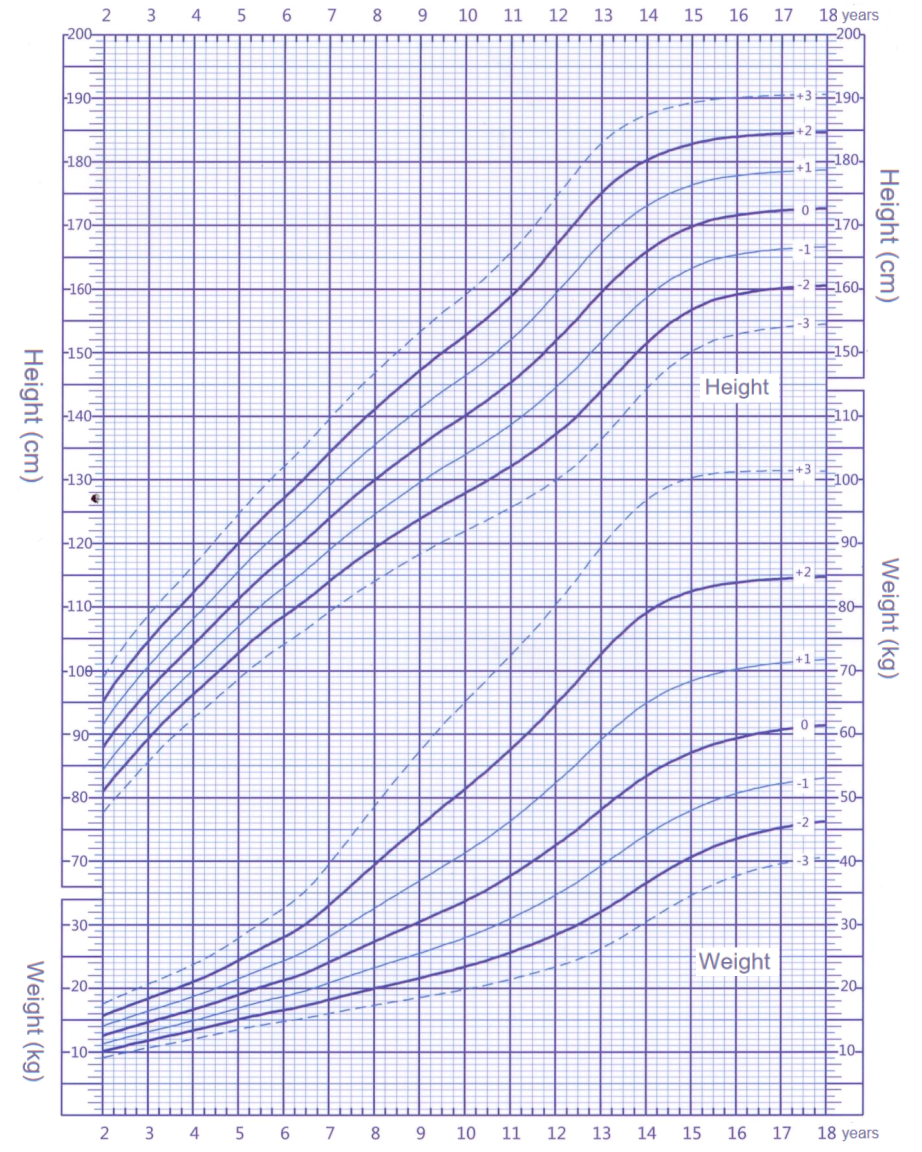


**Supplementary Figure 2.** Growth Curve Chart of Height and Weight Standard Deviation Units for Chinese Girls Aged 2–18 Years.


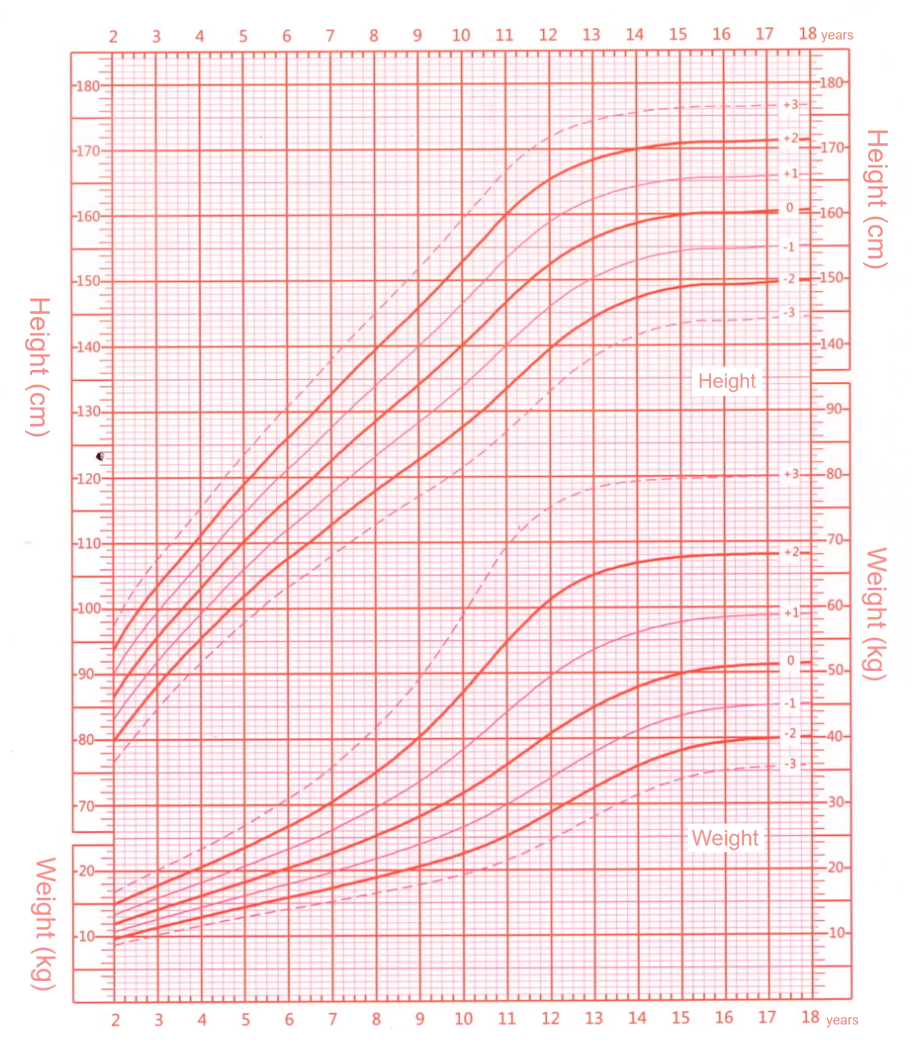

Supplement: Supplementary file 1 [file DataSheet1.docx]
